# Socio-spatial inequalities in accessibility of Indigenous community-controlled mental health services in South East Queensland, Australia

**DOI:** 10.1186/s12942-025-00415-9

**Published:** 2025-09-26

**Authors:** Lihong Zhang, Yan Liu, Lu Jin, Xiang-Yu Hou, Sandra Diminic, Xiaoyun Zhou, Shuichi Suetani, Carmel Nelson, Roxanne Bainbridge

**Affiliations:** 1 School of the Environment, The University of Queensland, Brisbane, QLD Australia; 2 Department of Geography and Resource Management, The Chinese University of Hong Kong, Shatin, Hong Kong SAR China; 3 School of Architecture, Design and Planning, The University of Queensland, Brisbane, QLD Australia; 4 Broken Hill University Department of Rural Health, Susan Wakil School of Nursing and Midwifery, The University of Sydney, Broken Hill, NSW Australia; 5 Queensland Centre for Mental Health Research, Brisbane, QLD Australia; 6 School of Public Health, The University of Queensland, Brisbane, QLD Australia; 7 Institute for Urban Indigenous Health, Brisbane, QLD Australia; 8 Queensland Brain Institute, The University of Queensland, Brisbane, QLD Australia; 9 School of Medicine and Dentistry, Griffith University, Gold Coast, QLD Australia; 10 Centre of Excellence for Indigenous Futures, The University of Queensland, Brisbane, QLD Australia

**Keywords:** Accessibility, Mental health services, Indigenous health, Socio-spatial inequality, South East Queensland

## Abstract

**Background:**

Mental disorders significantly burden Indigenous communities, worsened by limited culturally appropriate services. Spatial inequalities in access further disadvantage Indigenous peoples, especially in socio-economically challenged areas. This paper measures the spatial accessibility of Indigenous community-controlled mental health services in South East Queensland, Australia and examines its social inequalities across the region.

**Methods:**

We considered both population and health service providers’ capacity to maximise service coverage in measuring potential access to the services. Using Geographical Information Systems (GIS) technologies, a Gaussian-based two-step floating catchment area (G2SFCA) method was applied to quantify accessibility under four driving time thresholds ranging from 15 to 60 minutes. Bivariate global and local *Moran’s I* statistics were used to analyse social inequalities in accessibility across various geographical areas.

**Results:**

Accessibility was higher in urban areas than those towards the peri-urban and rural areas; the overall spatial coverage was relatively limited for service access within the 15- or 30-minute driving time threshold, compared with the 45- or 60-minute driving time threshold. Lower levels of accessibility were identified in areas with a concentration of Indigenous and socio-economically disadvantaged populations.

**Conclusions:**

This study advances a socially informed spatial inequality assessment framework. Unlike previous research exploring accessibility qualitatively, our framework innovatively integrates spatial analysis, Indigenous-specific population data and culturally sensitive provider capacity metrics within an advanced G2SFCA model. This approach uniquely exposes the compounded socio-spatial barriers to mental health services for Indigenous populations across South East Queensland’s urban-rural continuum. The resulting accessibility and inequality maps, combined with a summary of focus areas and their associated socio-demographic profiles, provide a direct policy lever to prioritise intervention for Indigenous communities experiencing the greatest disadvantage. By bridging spatial analysis with Indigenous cultural contexts, this work offers a replicable model for equitable, community-driven healthcare resource allocation for Indigenous peoples globally.

**Supplementary Information:**

The online version contains supplementary material available at 10.1186/s12942-025-00415-9.

## Introduction

Aboriginal and Torres Strait Islander (hereafter respectfully referred to as Indigenous) Australians have deep spiritual ties to specific geographical territories. Colonisation and the establishment of modern states displaced many Indigenous Australians to different territories [1]. Institutionalised racism and land dispossession have negatively impacted their social and emotional wellbeing, and the lasting effects of colonisation, intergenerational trauma, and socioeconomic disadvantage have resulted in poorer health outcomes [2]. Mental and substance use disorders are identified as the leading contributors to the disease burden in Indigenous communities and are exacerbated by high unmet mental health needs and limited access to services [3, 4]. Between 2009–10 and 2018–19, mental health-related hospitalisations among Indigenous Australians surged by 52%—significantly outpacing non-Indigenous populations—with particularly steep increases among Indigenous women (58%) [5]. Globally, Indigenous populations experience similar mental health inequities due to structural barriers including geographical isolation, lack of culturally inclusive care, and colonial legacies [6, 7]. Within Australia, stark regional disparities exist. For example, Indigenous youth in Victoria access mental health services at 1.4 times the rate of non-Indigenous youth, whereas those in the Northern Territory access services at half the rate [5]. Service gaps are most acute in remote areas, where public health service claims for specialist care among Indigenous Australians plummet to 161 per 1,000 people (vs. 860 in cities), with the rate 24–70% lower than non-Indigenous Australians and 30% reporting unmet healthcare needs due to distance or waiting times [8]. Addressing these disparities requires not only culturally safe care but also equitable spatial access to services, a dimension underexplored in existing research [9, 10]. Grounded in spatial equity theory [11], which emphasises the moral imperative to reduce geographical barriers to public resources, there is a trend of studies integrating health geography principles to examine the compounding effects of unequal service distribution on health disparities [12, 13].

Previous studies primarily rely on qualitative methods to explore how Indigenous populations access and use mental health services [14, 15]; spatial measures of the accessibility of mental health services (i.e., ease of access depending on the geographical location of potential patients) for Indigenous populations remain unexplored. Accessibility of mental health services can be understood as either the actual utilisation of services, referred to as realised access [16, 17], or the potential for access, in terms of service provision [18, 19]. As the ability to gain access is a prerequisite for the actual use of services, this paper focuses on *potential spatial accessibility*, that is, the relative ease for individuals to access services that are potentially available within a specified geographical area [20]. Although advanced spatial methods such as the two-step floating catchment area (2SFCA) model [21, 22] have been applied to measure spatial access to general health services, these studies face critical data limitations in Indigenous contexts [23]. Specifically, granular data on Indigenous population distribution, culturally specific service utilisation patterns, and provider capacity metrics (e.g., staffing, funding, service hours) are scarce or fragmented due to historical underinvestment and Indigenous data sovereignty concerns [24, 25]. These gaps hinder accurate modelling of accessibility, particularly for communities living in the peri-urban and rural areas.

Furthermore, Indigenous community-controlled health services (hereafter referred to as Indigenous services) are vital for mitigating barriers in accessing mental health services for Indigenous peoples. Evidence demonstrates that Indigenous services improve engagement and outcomes by centring cultural safety, a practice shown to reduce stigma, enhance trust, and align care with Indigenous worldviews [15, 26, 27]. For instance, studies associate Indigenous services with higher treatment adherence and lower hospitalisation rates for mental health conditions compared to mainstream services [28, 29]. Despite this, existing spatial analyses neglect Indigenous services as distinct providers, obscuring their role in addressing access inequalities.

Socio-economic disadvantage is also a factor in the accessibility of mental health services [30, 31]. Socio-economically disadvantaged groups typically face more barriers to accessing and using mental health services [32]. Lower levels of accessibility of community-level services contributes to dissatisfaction with the services, intensifies low-income groups’ perceived spatial deprivation, and hinders the formation of community attachment [33]. As such, it is crucial to investigate the degree of socio-spatial inequality in accessibility of mental health services for Indigenous populations at a neighbourhood scale [13].

This study aims to address two research questions: (i) How does the spatial accessibility of Indigenous community-controlled mental health services vary across South East Queensland (SEQ)? (ii) To what extent do socio-economic inequalities intersect with geographical disparities in accessibility? By applying a GIS-based method to map accessibility under various travel time thresholds and explore socio-spatial inequalities, this analysis provides policymakers with evidence to prioritise healthcare resources in spatial planning, reducing systemic barriers to culturally safe care, and advancing Indigenous populations’ mental health outcomes through place-based, Indigenous-led solutions.

## Literature review

### Accessibility of mental health services for Indigenous peoples

Research on Indigenous mental healthcare access has predominantly employed qualitative approaches to explore cultural barriers and systemic inequalities [34, 35]. While these studies illuminate critical demand-side challenges—such as distrust in mainstream services and staffing shortages in Indigenous community-controlled health services [36, 37]—they often neglect the spatial dimensions of accessibility [38]. Recent advancements in GIS-based spatial inequality frameworks enable a dual focus on geographical and social barriers [39–41], yet their application to Indigenous contexts remains sparse. For instance, a study in Canada’s Nunavik region exemplifies systemic gaps: despite Inuit self-governance, mental health services remain centralised in regional hospitals, forcing remote communities to rely on intermittent visits from external providers [42]. Similarly, another study shows that while Sámi cultural protocols are integrated into municipal health centres in Finnish Lapland, their day services for mental health are scarce outside urban hubs [42]. These international cases highlight how colonial legacies and decentralised governance exacerbate spatial inequalities, a pattern mirrored in Australia’s Kimberley region, where Indigenous community-controlled health services are critical but under-resourced in rural areas [42, 43].

A diverse range of quantitative spatial methods have been used to assess geographical accessibility of various health services. Statistical index methods assess the accessibility of health services via supply per capita (e.g., the quantity of full-time-equivalent psychiatrists and psychologists) within a specified areal unit [44]. However, these methods fail to offer insights into the nuanced spatial variations within a geographical unit. Spatial proximity methods investigate the minimum travel time or distance required to reach the nearest health services from residents’ home locations [17], the average travel time or distance to all health services, or the number of service providers that are reachable within an acceptable distance for patients [45]. T hese proximity measures, though, are somewhat unrealistic as they assume residents consistently opt for the nearest healthcare provider or there are no preferential differences between different healthcare providers [46]. The 2SFCA method and its variants (e.g., enhanced-2SFCA (E2SFCA), 3SFCA, inverted-2SFCA (i2SFCA)) [47–50], have revolutionised accessibility analyses by incorporating distance decay effects and population-weighted travel times [18]. However, their application to Indigenous populations is hindered by data scarcity, particularly the lack of granular Indigenous population distribution data and culturally specific utilisation metrics [23, 25]. For example, Canada’s Indigenous Services Agency struggles with fragmented data due to jurisdictional overlaps and historical underfunding [51], while Australia’s reliance on census units often obscures intra-regional disparities due to Indigenous data sovereignty concerns [52]. Recent studies in Canada using 2SFCA revealed clustered mental health services in urban cores, exacerbating disparities for peripheral neighbourhoods [53, 54]. However, the 2SFCA approaches have not been employed to measure mental health services accessibility for Indigenous peoples [19, 55, 56], a knowledge gap that this paper aims to fill.

### Socio-spatial inequalities in the accessibility of mental health services

As a limited public resource, health services, including mental health services, cannot be infinitely supplied and allocated with complete uniformity [20, 57], and so a key concern is whether certain geographical areas or socio-demographic groups have disadvantaged access to health services [58, 59]. Issues on the socio-spatial inequalities in health service access can be understood from both geographical and social dimensions, with both aspects being potentially intertwined [60, 61].

Mapping socio-spatial inequalities in health service access is crucial in understanding whether the socio-economically disadvantaged face more challenges in accessing health service resources. Analyses in Canada reveal persistent concentrated disadvantage in Métis communities, where poverty and transportation barriers compound with limited available services [62]. Tao and Cheng [63] explored the accessibility of health services amongst the elderly in Beijing and revealed that older adults face disadvantages in accessing health services compared with their younger counterparts. Kim et al. [64] discovered that neighbourhoods with a higher percentage of Latino or Hispanic residents in Florida, United States faced greater challenges in accessing health services during the COVID-19 period.

Socio-spatial inequalities in health service access are likely to stem from an uneven socio-economic distribution of the population, an uneven geographical distribution of healthcare providers, as well as the transport infrastructure that connects them [65]. While a growing body of literature explores spatial access to health services in general, few studies explore socio-spatial inequalities in the accessibility of mental health services [49]. Despite prior empirical research on the ways in which Indigenous peoples access and use mental health services [35, 66], socio-spatial inequalities in the accessibility of mental health services for Indigenous peoples is yet to be investigated using advanced spatial approaches.

## Methods

### Study context

As a fast-growing metropolitan region, SEQ has a population of approximately 3.8 million, encompassing 12 Local Government Areas (LGAs). According to the 2021 Census of Population and Housing, 109,539 people residing in SEQ identify themselves as Indigenous Australians, accounting for 2.91% of the total population in SEQ, and 11.1% of Australia’s Indigenous population [67]. Five of the 12 LGAs (Brisbane, Moreton Bay, Logan, Gold Coast and Ipswich) collectively have the largest Aboriginal and Torres Strait Islander populations in Queensland, comprising of 34.6% of the total Aboriginal and Torres Strait Islander populations in Queensland [68]. The local tribes of the area include the Jagara/Yuggera, the Yugambeh/Yugumbir, the Quandamooka/Nunukul, the Gubbi Gubbi/Kabi Kabi, the Minjungbal/Minyangbal, the Butchulla/Batjala, the Waka Waka, and the Goinbal peoples [69].

The shortage in mental health services in the SEQ region as a whole, and even more so for Indigenous Australians, has been identified as a priority issue in the Health Workforce Strategy for Queensland [70]. From 2009–10 to 2018–19, the hospitalisation rate relating to mental health conditions for Indigenous Australians increased by 52%, from 19 to 29 per 1000 hospitalisations [71]. In addition, since the 1980s, SEQ has witnessed a notable rise in Indigenous migration towards urban areas, a perpetuation of displacement away from ancestral homes, and projections suggest that by 2031, the population in the urban SEQ region will surpass 130,000 people, an increase of almost 20% from 2021 (Brand et al., 2016). Considering the growing Indigenous population and the shortage of mental health services in SEQ, there is a pressing need to quantify the spatial accessibility of mental health services and how this varies in relation to socio-demographic characteristics [72].

### Data sources

Datasets used encompassed population and socio-demographic data, mental health service data, and transportation network data. Population data was sourced from the 2021 Census of Population and Housing at the Statistical Area Level 2 (SA2) [67]. As the SA2 unit approximates to the scale of a suburb or community,^1^ we considered this scale suitable for capturing the distribution of Indigenous populations with relatively high data reliability.^2^ We defined the demand locations of Indigenous populations using the weighted mean centre of each SA2,^3^ with the weight being defined by the number of Indigenous Australians at the Statistical Area Level 1 (SA1).^4^

**Fig. 1:**
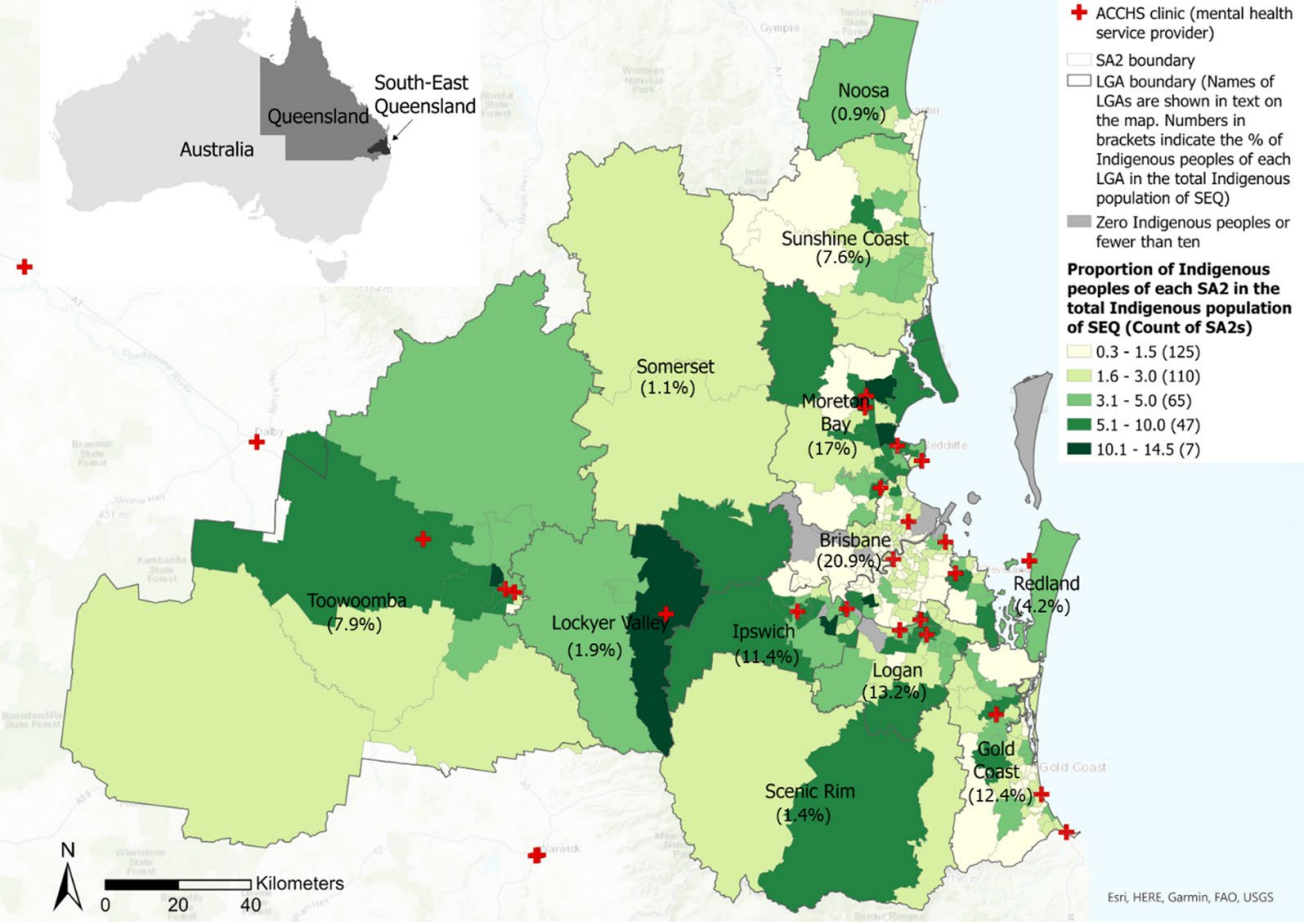
The study area and its Indigenous population distribution

We also retrieved data on mental health service providers from the Australian Indigenous HealthInfoNet [77], an online platform funded by the Australian Government Department of Health and Aged Care that provides open access data and resources to guide practice and policy in Aboriginal and Torres Strait Islander health. We focused on the health or medical clinics of Aboriginal Community Controlled Health Services (ACCHSs) , which are also known as Aboriginal and Islander Community Controlled Health Services, Aboriginal and Torres Strait Islander Community Health Services, or Regional Aboriginal and Islander Community Controlled Health Organisations. ACCHSs are run by organisations governed by local Indigenous communities through elected management boards (as opposed to State governments), and deliver primary healthcare services, including mental health services, to Indigenous Australians. We accessed the database in November 2023. Data collected include the clinic name, address, and number of health professionals. We further verified the data by inquiring by phone and visiting official webpages of each clinic, where possible. In calculating the number of health professionals, we included general practitioners, mental health professionals (i.e., counsellors, psychologists and psychiatrists), and Indigenous health professionals (i.e., Indigenous health practitioners and Indigenous health workers), since all three types of health professionals are usually working as a team to provide mental health services.^5^ Utilising the geocoding tool in ArcGIS Pro 3.1, we mapped the geographical locations of the ACCHS clinics that provide mental health services (see Fig. 1). Considering that administrative boundaries can distort measured healthcare availability for nearby residents, as they may live closer to facilities across the boundary than within it [78], we included ACCHS clinics and Indigenous demand locations within a 100-kilometre threshold to align with the largest service catchment area within a 60-minute driving threshold (see the *Analytical Approaches* section for details). This adjustment helps minimise the border effect when calculating potential accessibility in border areas [78]. However, due to data limitations, this buffer zone does not extend beyond the New South Wales border. The impact of this exclusion is minimal, as cross-state healthcare service utilisation is primarily driven by demand for hospitals rather than healthcare clinics [79].

While ACCHSs are foundational to culturally safe healthcare for Indigenous Australians, we acknowledge that not all Indigenous individuals access these services exclusively. People may choose to utilise mainstream health services due to geographical, logistical, or personal preferences, which may differ in their capacity to provide culturally safe care [80]. However, our study focuses on ACCHS as they are uniquely governed by Indigenous communities and explicitly prioritise cultural safety as a core component of service delivery [27]. Nevertheless, the absence of direct user perspectives on cultural safety within the dataset represents a limitation. ACCHS administrative data, while critical for mapping spatial accessibility, does not capture qualitative measures of cultural safety, such as patient experiences, trust in providers, or alignment with Indigenous healing practices.

Furthermore, we collected socio-demographic data from the Australian Bureau of Statistics (ABS) 2021 Census at the SA2 level, with indicators including the proportion of Indigenous peoples in each SA2 in the total Indigenous population in SEQ (referred to as the Indigenous population proportion hereafter), the Index of Relative Socio-economic Advantage and Disadvantage (IRSAD) and the Indigenous Relative Socio-Economic Outcomes (IRSEO) [81, 82]. The IRSAD represents the economic and social circumstances of individuals and households within each SA2 for the total population in Australia. Research has shown that socio-economic disadvantages contribute to reduced utilisation of mental health services [31] and can influence Indigenous Australians’ experience in accessing mental health services [35]. A higher IRSAD score signifies a relative absence of disadvantage or higher level of advantage overall, and vice versa (see Fig. A in Additional File 3 for the distribution of IRSAD scores). In contrast, the IRSEO measures the relative socio-economic characteristics of Indigenous Australians residing in different regions [83]. Given the relatively small size of Indigenous populations and a consistently lower socio-economic status across the country, their socio-economic characteristics might differ from their non-Indigenous counterparts [84, 85]. A higher IRSEO score indicates a comparatively higher level of disadvantage, and vice versa (see Fig. B in Additional File 3 for the distribution of IRSEO scores). Examining both the IRSAD and IRSEO enables an understanding of areal socio-economic disadvantages from the perspectives of both the total population and the Indigenous population, respectively. Doing this contributes to a holistic understanding of the socio-economic environment for Indigenous Australians residing in each SA2.

**Fig. 2:**
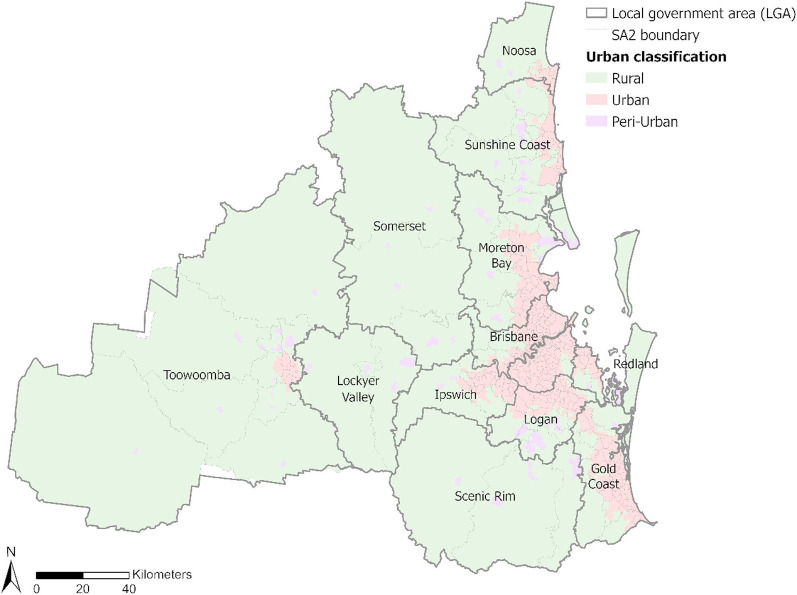
Spatial distribution of the urban classification based on the SOS framework

To assess accessibility variation across the urban-rural continuum, we classified the study region into three categories—*urban*,* peri-urban*, and *rural*—using the Section of State (SOS) framework from ABS [86]. SOS is based on Urban Centres and Localities (UCLs), which are defined by dwelling and population density using the 2021 Census data. We mapped the SOS categories as follows: *Major Urban* corresponds to *urban*, which refers to urban centres with a population exceeding 100,000; *Other Urban* corresponds to *peri-urban*, representing urban centres with a population ranging from 1,000 to 99,999, which are transitional zones between urban and rural, sharing characteristics of both urban and rural landscapes [87]; *Bounded Localities* and *Rural Balance* correspond to *rural*, with the former being population clusters of 200 to 999 inhabitants, and the latter comprising the remaining non-urban land area within SEQ. Since UCLs are aggregated from SA1s, which are smaller than SA2s, the resulting urban classification boundaries do not fully align with SA2 boundaries. The spatial distribution of these classified areas is presented in Fig. 2.

We also collected road network data from OpenStreetMap (OSM) [88] to calculate the travel time by driving between neighbourhoods and healthcare providers using the Python package, OSMnx [89]. As OSM data contains information on road types, connectivity, maximum travel speed and road length, we were able to identify driving routes with the least travel time between origins and destinations. We used driving as the travel mode since approximately 88% of Indigenous households in Australia have at least one motor vehicle, according to the latest census [90]; other modes of transport such as walking and cycling are generally unrealistic and/or inconvenient for accessing mental health service providers, particularly in low-density suburban and rural areas in the Australian context. It is important to note that while some patients may not have private vehicles, the provision of vehicular transport in the form of clinic-based pick-up services is a key service of ACCHS clinics. We consider the travel time using such transport services as equivalent to driving a private vehicle.

### Analytical approaches

#### Quantifying mental health service accessibility

To quantify the supply of mental health services in measuring accessibility, we calculated the mental health service capacity ($\:{C}_{i})$ of ACCHS clinics, using a weighted index adapted from Zhang et al. [91]:

$$\:{C}_{i}\:=\:{GP}_{i}\times\:0.4+{MP}_{i}\times\:0.4+{IP}_{i}\times\:0.2$$

where, $\:{C}_{i}$ refers to the mental health service capacity of clinic $\:i$; $\:{GP}_{i}$, $\:{MP}_{i}$, and $\:{IP}_{i}$ denote the counts of general practitioners (GPs), mental health professionals (MPs), and Indigenous health professionals (IPs) at clinic $\:i$, respectively. The weighting scheme reflects workforce contributions to direct clinical care, guided by ACCHS operational models [92] and national mental health workforce standards [93]. GPs and MPs were assigned equal weights (0.4) due to their central roles in diagnosing, treating, and managing mental health conditions (i.e., tasks that typically dominate patient interactions). For example, in ACCHS clinics in SEQ such as Moreton Aboriginal and Torres Strait Islander Community Health Service (MATSICHS) in Strathpine and ATSICHS Brisbane in Logan, GPs account for 60–70% of primary mental health consultations, while MPs deliver specialised interventions (e.g., trauma-informed counselling, suicide prevention programs). IPs were assigned a weight of 0.2 in the health service capacity ($\:{C}_{i}$) calculation. This weighting reflects the fact that, while their work is critical for mitigating systemic barriers to care—notably the historical and cultural distrust of mainstream services prevalent in many Indigenous communities [94]—IPs often supplement clinical care through cultural brokerage, community engagement, and holistic support (e.g., facilitating access to traditional healing and coordinating social services) [95]. For instance, IPs at the Kalwun Miami health clinic lead yarning circles and cultural mentorship programs, which strengthen trust but are less directly tied to clinical throughput. However, we recognise that this weighting may underrepresent IPs’ systemic impact on long-term health outcomes. 

Annual report of the ACCHS Network further informed this structure: in ACCHS clinics in SEQ run by the Institute for Urban Indigenous Health (IUIH) in 2023, GPs and MPs collectively managed approximate 80% of mental health and substance use related cases [96]. IPs, in contrast, support broader determinants of wellness, aligning with their lower clinical weight but reinforcing the need for complementary qualitative assessments of their impact [97].

We employed a Gaussian-based 2SFCA (G2SFCA) method to calculate the spatial accessibility of mental health services at ACCHS clinics across SEQ. Building on the standard 2SFCA method—which assumes binary accessibility within fixed catchment zones—the G2SFCA method enhances this approach by incorporating a continuous distance decay function (Gaussian kernel) to weight service availability based on travel time, better reflecting real-world travel behaviour where accessibility diminishes gradually with distance [98, 99]. To evaluate sensitivity to catchment size, we tested four driving time thresholds (15, 30, 45, and 60 minutes), with the largest threshold approximating a 100-kilometre service radius (see Additional File 1 for methodological details).

The G2SFCA method strikes a balance between computational simplicity and realism compared to alternative methods. Unlike simpler 2SFCA models, it avoids abrupt accessibility cutoffs, which are particularly problematic in rural/peri-urban SEQ where sparse clinic distribution amplifies sensitivity to distance assumptions. However, the G2SFCA method assumes a symmetric Gaussian distance decay effect, which may oversimplify actual travel patterns (e.g., directional road network constraints or Indigenous-specific mobility preferences). While agent-based or network-optimised models could capture these nuances, such approaches require granular individual mobility data that is unavailable in this study. The G2SFCA’s reliance on aggregate travel times and population locations aligns with our datasets, enabling scalable analysis across urban-rural gradients.

This method is particularly suited to addressing our research questions. First, its capacity to model continuous accessibility gradients allows us to map subtle disparities across SEQ’s heterogeneous geography (urban, peri-urban, rural), directly addressing our first research question. Second, by integrating spatially explicit socio-economic data with accessibility outputs, the G2SFCA method facilitates the identification of overlapping geographical and equality barriers (our second research question), a task less feasible with purely binary accessibility metrics. Though the Gaussian decay parameter introduces some arbitrariness, our multi-threshold sensitivity analysis mitigates this limitation, ensuring robustness in identifying systemic inequalities.

#### Measuring socio-spatial inequalities in accessibility

To explore the socio-spatial inequalities in accessibility, we used the bivariate global *Moran’s I* [100] and the bivariate local *Moran’s I* statistics [101]. The bivariate global *Moran’s I* assesses the overall spatial correlation between accessibility and each of the socio-demographic factors across the entire study area; however, it may overestimate spatial autocorrelations relating to the inherent correlation of accessibility and the socio-demographic factors [100]. The bivariate local *Moran’s I* measures the local spatial relationships and evaluates whether the value of accessibility at one SA2 is associated with the values of a socio-demographic factor at its neighbouring SA2s. The results are presented using bivariate Local Indicator of Spatial Association (LISA) maps. Details of the methods used are provided in Additional File 2.

## Results

### Accessibility of mental health services from ACCHS clinics in SEQ

**Fig. 3:**
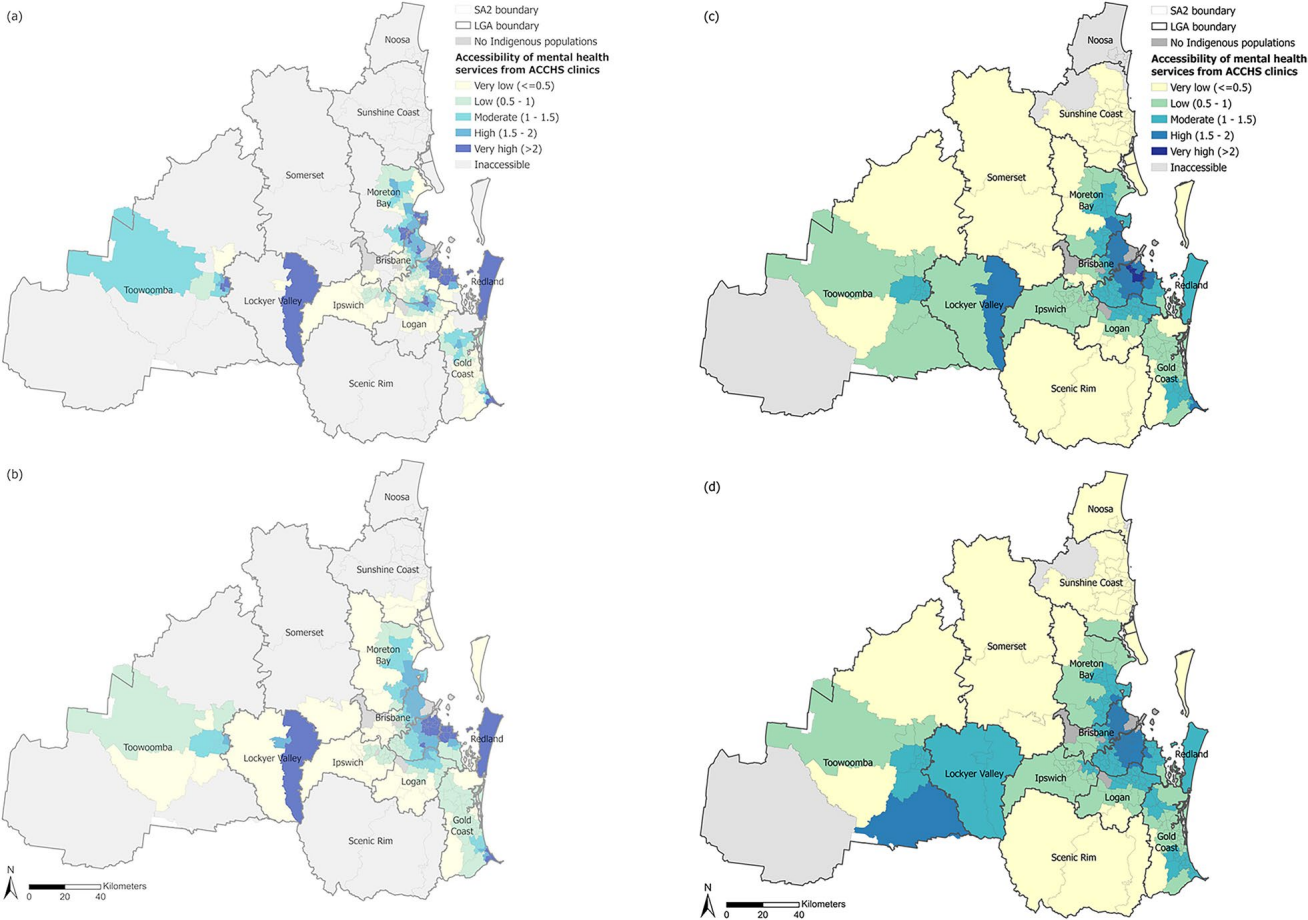
Accessibility of mental health services from ACCHS clinics for Indigenous Australians within (**a**) 15-minute, (**b**) 30-minute, (**c**) 45-minute and (**d**) 60-minute driving time thresholds

We classified accessibility under each of the four driving time thresholds (i.e., 15, 30, 45 and 60 minutes) into five levels (i.e., very low, low, moderate, high, and very high), using a classification method based on Natural Breaks (Jenks) and adjusted manually to maintain consistency across different driving time thresholds, as shown in Fig. 3. Overall, Indigenous Australians living in the urban area of the LGAs of Brisbane, Gold Coast, Logan, Moreton Bay, Redland, Lockyer Valley and Toowoomba have moderate to very high access to mental health services from ACCHS clinics under all four driving time thresholds. For each threshold, accessibility tends to recede outwards following a radial pattern from very high and high to lower levels of accessibility (Fig. 3). Generally, accessibility is higher in urban areas than locations towards the peri-urban and rural areas, especially under the 15-minute and 30-minute scenarios. Within the 15-minute driving threshold, 64 out of 346 SA2s (excluding those with zero Indigenous Australians) have no access to an ACCHS clinic, while under a 60-minute driving threshold, only four SA2s have no access. Furthermore, under the longer driving thresholds (45 and 60 min), despite fewer SA2s having no access to any ACCHS clinic, there are also fewer SA2s with very high values of accessibility. The reason for which an SA2 can have different accessibility levels at different driving thresholds is that accessibility values are determined by both service supply and service demand. This implies that although a greater driving threshold means more healthcare providers may be available, the services are also shared with a larger population who can also access these healthcare providers within the same driving time.

### Socio-spatial inequalities in accessibility

Table 1 presents the correlation between accessibility and the socio-demographic factors in SEQ from the bivariate global *Moran’s I* statistic. The results show that under the driving time thresholds of 30, 45, and 60 minutes, the relationships between accessibility and the respective socio-demographic factors are all statistically significant (*p* < 0.010). For the Indigenous population, accessibility under the 30- to 60-minute driving time thresholds has a negative relationship with Indigenous population proportion, indicating that areas with low levels of accessibility are generally near those with high Indigenous population proportions in the region. This indicates that socio-spatial inequalities tend to increase when considering service catchment areas larger than a 15-minute drive in SEQ. The positive relationship with IRSEO indicates that areas with high levels of accessibility are surrounded by high levels of Indigenous-specific socio-economic disadvantage. This suggests that the deployment of mental health services from ACCHS clinics has somewhat addressed the needs of socio-economically disadvantaged neighbourhoods within Indigenous communities. However, the positive relationship with IRSAD signals that areas with low levels of accessibility are surrounded by those with high levels of socio-economic disadvantage for the total population. This implies that, as part of the total population in each SA2, Indigenous residents may still be subject to socio-spatial inequalities in accessing mental health services from ACCHS clinics. Conversely, accessibility within a 15-minute driving time threshold is less significantly correlated with the Indigenous population proportion (*p* < 0.05) and IRSAD (*p* < 0.1) with the directions of the correlations opposite to those of other thresholds. This may be due to more areas having zero accessibility within this shorter driving time. Overall, socio-spatial inequalities in access to mental health services from ACCHS clinics are more pronounced when considering a driving time threshold higher than 15 minutes.

**Table 1:** Bivariate global *Moran’s I* between accessibility and socio-demographic factors for SEQ

Accessibility	Socio-demographic factors		
	Indigenous population proportion	IRSEO score	IRSAD score
Within a 15-min driving time	0.060 **	0.017	− 0.031 *
Within a 30-min driving time	–0.090 ***	0.134 ***	0.172 ***
Within a 45-min driving time	–0.166 ***	0.164 ***	0.263 ***
Within a 60-min driving time	–0.161 ***	0.111 ***	0.254 ***

* *p* < 0.1; ** *p* < 0.05; *** *p* < 0.01

The bivariate LISA maps reveal a gradual, albeit subtle, change in areas characterised by certain types of spatial correlations between accessibility and socio-demographic factors across all four driving time thresholds, which are discussed further below.

#### Accessibility relative to the Indigenous population proportion

**Fig. 4:**
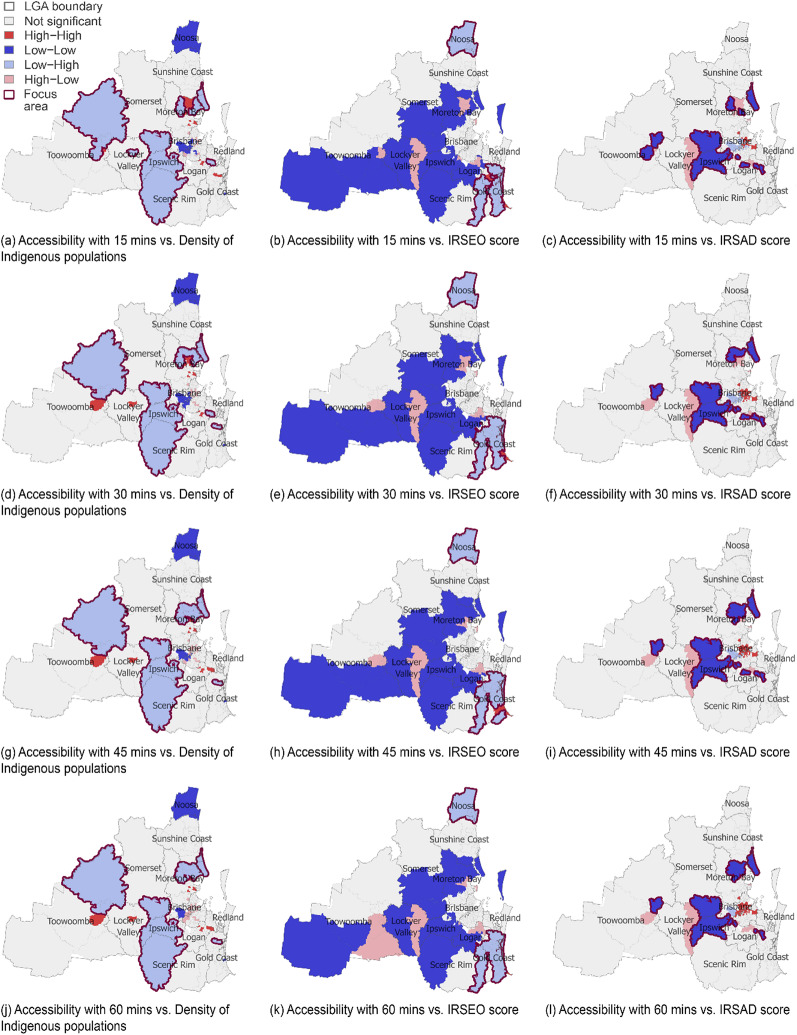
The bivariate LISA maps between accessibility and socio-demographic factors

High-High clusters (i.e., SA2s with relatively high levels of accessibility clustered with those with relatively high Indigenous population proportions) are mainly located in the urban areas of Moreton Bay, Logan, Lockyer Valley and Toowoomba, indicating the least socio-spatial inequality in accessing mental health services from ACCHS clinics for Indigenous Australians in those areas. Low-High clusters (i.e., SA2s with relatively low levels of accessibility clustered with those with relatively high Indigenous population proportions) are mostly found in the suburban areas of Moreton Bay, Somerset, Ipswich, Scenic Rim and Toowoomba; these areas exhibit socio-spatial inequalities between the distribution of accessibility and the proportion of Indigenous Australians. These results are shown in Fig. 4 (a), (d), (g) and (j), where SA2s with the most significant socio-spatial inequalities are labelled as Focus Areas. This labelling convention is applied to all maps in Fig. 4.

#### Accessibility relative to the IRSEO score

High-Low clusters (i.e., SA2s with relatively high levels of accessibility clustered with those with relatively low levels of Indigenous-specific socio-economic disadvantage) are mainly located in the urban areas of Moreton Bay, Logan, Lockyer Valley and Toowoomba. Low-High clusters (i.e., SA2s with relatively low levels of accessibility and relatively high levels of Indigenous-specific socio-economic disadvantage) are identified in Noosa, the suburban areas of Gold Coast and the eastern parts of the Scenic Rim. Low-High clusters exhibit the greatest socio-spatial inequalities with respect to the IRSEO score, and the relevant SA2s show little overlap with those exhibiting the greatest socio-spatial inequalities with respect to the IRSAD score. These results are shown in Fig. 4 (b), (e), (h) and (k).

#### Accessibility relative to the IRSAD score

High-High clusters (i.e., SA2s with relatively high levels of accessibility clustered with those with relatively high IRSAD scores) are mainly located in the central and eastern urban areas of Brisbane under all four driving time thresholds, implying that these areas have relatively good accessibility to mental health services from ACCHS clinics and are surrounded by areas where residents are relatively socio-economically advantaged. High-Low clusters (i.e., SA2s with high levels of accessibility and low IRSAD scores) are present in the urban areas in the Moreton Bay, Logan, Lockyer Valley and Toowoomba LGAs, indicating that areas with relatively high levels of accessibility are clustered with areas that are relatively socio-economically disadvantaged. Low-Low clusters (i.e., SA2s with low levels of accessibility and low IRSAD scores) are concentrated in specific suburban or regional areas in the Moreton Bay, Logan, Somerset, Ipswich and Toowoomba LGAs, indicating relatively low levels of accessibility and relatively high levels of socio-economic disadvantage. The low-low clusters disclose the greatest socio-spatial inequalities in accessibility. These results are shown in Fig. 4 (c), (f), (i) and (l).

## Discussion and conclusions

This study assesses the accessibility of mental health services for Indigenous populations and explores its socio-spatial inequalities in SEQ. Our measure of accessibility focuses on potential access based on entire Indigenous populations—rather than existing Indigenous patients who have used mental health services (i.e., realised access)—to account for the coverage capacity of health service providers and uses data on ACCHS clinics to represent the available culturally appropriate mental health services. It is worth noting that all measures are relative and do not necessarily indicate sufficient accessibility in absolute terms. Our framework innovates by integrating Indigenous population data and culturally sensitive provider capacity weightings into an advanced G2SFCA model. Integrating spatial modelling with critical socio-demographic determinants provides a more accurate and actionable assessment of health inequalities for Indigenous populations.

The results show that urban areas have better access compared with locations further towards the peri-urban areas and in rural hinterlands, especially within a 15- or 30-minute driving time threshold. This urban-rural gradient highlights systemic inequalities in service distribution, where densely populated urban cores in the Brisbane and Gold Coast LGAs benefit from concentrated ACCHS resources even though these areas may not necessarily have high concentration of Indigenous peoples, while dispersed populations in peri-urban and rural regions face compounded barriers due to distance and under-resourcing. Areas with low or zero accessibility are also identified in remote pockets of SEQ where Indigenous communities may rely on outreach programs or telehealth, but these modalities are not captured in our spatial analysis.

The results also demonstrate how accessibility may vary across a region relative to socio-demographic factors, and we identify areas showing socio-spatial inequalities. Global statistics reveal that areas with lower levels of accessibility tend to be surrounded by those with higher proportions of Indigenous populations, while areas with higher levels of accessibility tend to be adjacent to those experiencing higher levels of Indigenous-specific socio-economic disadvantage. This mismatch suggests that ACCHS placement in SEQ partially prioritises areas with acute Indigenous-specific socio-economic needs but fails to address regions where Indigenous populations are concentrated but underserved. However, lower-accessibility areas also tend to border areas with greater socio-economic disadvantage affecting the entire population, including Indigenous communities. This overlap implies that systemic deprivation in marginalised neighbourhoods—urban or rural—intersects with Indigenous health service inequalities, necessitating place-based interventions tailored to local demographic and geographical contexts. For each socio-demographic factor, local socio-spatial inequalities in accessibility evolve gradually and subtly across the four driving time thresholds. This result demonstrates the robustness of our socio-spatial inequality analysis in terms of accessibility measures across the tested driving time thresholds. The socio-spatial inequality findings align with prior qualitative research on the socio-economic barriers for Indigenous populations in accessing mental health services [35, 102] and goes further to explicitly identify neighbourhoods where relatively low accessibility of culturally appropriate mental health services coincides with relatively high Indigenous population proportions and low socio-economic status.

To translate the resulting maps into straightforward policy levers, we have synthesised the identified focus areas into a table (see Additional File 4), summarising key geographical units—SA2s—with the most socio-spatial inequalities, pairing the calculated accessibility scores with demographic and socioeconomic profiles. By identifying focus areas for priority actions, this study provides actionable insights for policymakers to optimise mental health service delivery and address socio-spatial inequalities via targeted policy intervention and resource allocation strategies. For example, government departments for health and transport could work together in optimising service catchments to ensure services align with cultural expectations of acceptable travel burdens [31]; co-location of services such as embedding ACCHS mental health teams within Centrelink offices, schools, or housing services to reduce systemic barriers in accessing mental health services for communities facing multi-dimensional deprivation [35]; and areas identified to be considered for ACCHS funding allocation to ensure resources align with both geographical need and socio-cultural equality principles [96].

The framework developed here provides a replicable model for assessing mental health service accessibility for Indigenous populations in other regions and countries. The methodology of integrating advanced spatial modelling with culturally sensitive metrics is directly transferable to other Indigenous contexts (e.g., Canada, New Zealand, the United States). Researchers can adapt our model by incorporating local data on Indigenous population distribution, culturally appropriate services, and community-defined travel parameters. Furthermore, the core findings—such as the urban-rural access gradient and the socio-spatial inequalities—reflect systemic issues likely present elsewhere, making our approach a valid tool for international comparison and targeted intervention. Successful application, however, requires contextual adaptation of parameters (e.g., specific catchment sizes, capacity weightings, and socio-demographic variables), data availability, and community partnership to ensure cultural safety and relevance.

This study has several limitations that warrant further consideration in future research. First, due to limited data availability, accessibility was assessed for private vehicle travel, and excluded public transport, walking, or community-provided transport, which could be important for Indigenous Australians [103, 104]. Second, the accuracy and completeness of data sources (e.g., OpenStreetMap road networks, ACCHS clinic locations) and inherent limitations in Local Moran’s I, could potentially affect our findings. For instance, OSM data can underrepresent rural road conditions, while ACCHS service descriptions may not fully capture on-the-ground operational capacities and do not include mainstream services.

Future research could employ advanced methods (e.g., data disaggregation techniques) to produce granular data and perform sensitivity analyses to address scale-dependent biases in accessibility assessments. It would be ideal to engage with Indigenous communities to co-design accessibility metrics, integrating Indigenous knowledge systems (e.g., mobility patterns, cultural site significance) into spatial analyses using qualitative metrics (e.g., client-reported impacts of various health professionals) to better reflect lived experiences in accessibility measurement. Methodologically, we could also consider integrating commuting flows, activity space trajectories, and temporal service data to model accessibility as a composite of locations people frequently visit, not just their homes, which would better expose inequalities—such as gaps along commuter routes—and advance mobility-aware models to foster spatially responsive interventions.

In conclusion, this study provides scientific evidence for future spatially-focused policy and interventions to improve service resource provision and allocation, helping to close the gap in mental health care access for Indigenous Australians. The application of these methods for assessing accessibility and socio-spatial inequalities to other Indigenous groups around the world has the potential to deepen the understanding of mental health care access for Indigenous populations at a more context-specific level.

## Supplementary Information

Additional file 1: Description of the Gaussian-based two-Step Floating Catchment Areamethod. Details of the G2SFCA method employed in this study, including accompanying text and equations

Additional file 2: Description of the bivariate global *Moran’s I* and bivariate local *Moran’s I* statistics. Details of the bivariate global *Moran’s I* and bivariate local *Moran’s I *statistics, including their application in this study.

Additional file 3: Spatial distribution of the socio-economic disadvantage in SEQ. Maps of the 2021 IRSAD and IRSEO score distributions by SA2 across SEQ

Additional file 4: Focus areas for prioritising Indigenous community-controlled mental health service development in SEQ. The summary table of identified focus areas by SA2, alongside their socio-demographic characteristics

## Data Availability

All datasets analysed or generated during this study are available within this published article.

## Abbreviations

SEQ: South East Queensland
LGA: Local Government Area
SA2: Statistical Area Level 2
OSM: OpenStreetMap
G2SFCA: Gaussian-based two-step floating catchment area
GP: General practitioner
E2SFCA: Enhanced two-step floating catchment area
2SFCA: Two-step floating catchment area
LISA: Local Indicator of Spatial Association
IRSAD: Index of Relative Socio-economic Advantage and Disadvantage
IRSEO: Indigenous Relative Socio-economic Outcomes
ACCHS: Aboriginal Community Controlled Health Service
ATSICHS: Aboriginal and Torres Strait Islander Community Health Service
ABS: Australian Bureau of Statistics
IUIH: Institute for Urban Indigenous Health
NHMRC: National Health and Medical Research Council

## Footnotes

1 SA2s represent functional zones that act as communities, engaging in economic and social interactions within major urban areas of Australia [73]. Typically, SA2s have a population ranging from 3,000 to 25,000 people, with an average population size close to 10,000 people [74]. Previous empirical research in urban studies have used SA2s as neighbourhoods’ functional equivalent [75].

2 We also tested using Statistical Area Level 1 (SA1), a smaller geographical unit than SA2, as the spatial unit to retrieve the Indigenous populations data from the 2021 census TableBuilder data platform. However, a large number of SA1s (56.3%) had a very small number of Indigenous Australians at this spatial unit. According to ABS, when a small data number (i.e., single digits) is extracted from the TableBuilder, a perturbation technique is applied to randomly adjust the data value instead of exporting the original data, to mitigate the risk of identifying individuals in aggregate statistics, making the exported data unreliable [76]. As such, the SA2 unit is more suitable, and the data extracted is more reliable for further analysis in this study.

3 Within the study area, eight SA2s have zero Indigenous residents, and one SA2 has nine people of which the accuracy could be influenced by the perturbation technique [76]. Caution should go with inferences regarding the accessibility results for these SA2s.

4 SA1 is the second smallest unit for the release of census data, with each SA1 having a population ranging from 200 to 800 persons in general and an average of about 400 persons; it provides population data at a smaller scale and therefore can be applied to define the population-weighted mean centre of SA2s.

5 General practitioners have been included since they are the first point of contact for patients seeking mental health support, they provide general health care and interventions for common mental health issues, and they are required for Medicare-subsidised sessions for secondary and tertiary mental health services in Australia. Indigenous health practitioners deliver clinical services and patient care, focusing on culturally safe practices for Indigenous Australians. Indigenous health workers assist with the coordination and provision of health care delivery to Indigenous communities.
