# Supplementary material for: Socio-spatial inequalities in accessibility of Indigenous community-controlled mental health services in South East Queensland, Australia
Source: Int J Health Geogr. 2025 Sep 26;24:24. doi: 10.1186/s12942-025-00415-9 (PMC12465140; doi:10.1186/s12942-025-00415-9)
Supplement: Supplementary file 1 — Additional file 1: Description of the Gaussian-based two-Step Floating Catchment Areamethod. Details of the G2SFCA method employed in this study, including accompanying text and equations [file 12942_2025_415_MOESM1_ESM.docx]

**Additional File 1: Description of the Gaussian-based two-Step Floating Catchment Area (G2SFCA) method**

The G2SFCA method is based on the 2SFCA method [1, 2]. The 2SFCA method comprises two steps. In the first step, the supply-to-demand ratio is calculated at each health service provider (i.e., ACCHS clinics) location by accounting for all population centres (i.e., Indigenous population weighted mean centres of SA2s) located within the catchment area of the health service provider (Eq. 1). The second step involves assigning health service providers to the population centre by identifying all health service providers that fall within the catchment area of each population centre. Subsequently, the supply-to-demand ratio calculated in the previous step is summed for each population centre (Eq. 2). We then introduced a Gaussian function (Eq. 3) to consider the distance decay effect in both Eq. 1 and Eq. 2, assuming that the potential accessibility of health services at a given population centre is equivalent to the sum of the distance-decay-weighted supply-to-demand ratios for all health service providers located within the catchment area of the population centre. We used a Gaussian function as it has been demonstrated to outperform other functions in replicating the distance decay effect [3]. The distance decay coefficient, $\beta$, is a critical parameter in the G2SFCA method as it governs the rate at which access to a service diminishes with increasing travel time (Eq. 4) [4, 5]. Rather than being an arbitrarily selected value, β was calculated directly from the predetermined service catchment size ($d_{0}$) to ensure a mathematically consistent and theoretically sound decay function. This calculation is based on the principle of defining an effective catchment area. Following the established methodology of Kwan [6], we set a critical threshold value of 0.01 for the Gaussian function $G(d_{\begin{aligned} &ij \\ & \end{aligned}} , d_{0})$. This threshold signifies that locations at the maximum catchment boundary ($d_{\begin{aligned} &ij \\ & \end{aligned}}=d_{0}$) are assigned a negligible weight, effectively meaning access from beyond this boundary is not considered. The derivation of β is achieved by solving the Gaussian function at the boundary condition where $d_{\begin{aligned} &ij \\ & \end{aligned}}=d_{0}$ and $G(d_{\begin{aligned} &ij \\ & \end{aligned}} , d_{0})$=0.01. Starting with the standard form of the Gaussian decay function (Eq. 3), we substitute the boundary values with 0.01 to get 0.01 = $e^{- {{d_{\begin{aligned} &0 \\ & \end{aligned}}}^{2}}/\beta}$. To solve for β, we take the natural logarithm ($Ln$) of both sides and get $Ln(0.01)$ = $- ({{d_{\begin{aligned} &0 \\ & \end{aligned}}}^{2}}/{\beta)}$. Rearranging the previous equation to isolate β yields the final formula as Eq. 4.

$$R_{j}= \frac{S_{j}}{\sum_{k\in\left\{ d_{kj}\leq d_{0} \right\}} D_{k}G\left( d_{kj}, d_{0} \right)} \left( 1 \right)$$

$$A_{i}= \sum_{j\in\left\{ d_{ij}\leq d_{0} \right\}} R_{j}G\left( d_{ij}, d_{0} \right) \left( 2 \right)$$

$G\left( d_{\begin{aligned} &ij \\ & \end{aligned}} , d_{0} \right)= e^{- \frac{{d_{\begin{aligned} &ij \\ & \end{aligned}}}^{2}}{\beta}}= e^{\frac{{d_{\begin{aligned} &ij \\ & \end{aligned}}}^{2}Ln0.01}{{d_{0}}^{2}}} \left( 3 \right)$

$\beta= -\frac{{d_{0}}^{2}}{Ln(0.01)} \left( 4 \right)$

where:

R_j_ is the supply-to-demand ratio of health service provider j;

S_j_ is the supply capacity of mental health services by health service provider j;

D_k_ is the demand at population centre k falling within the catchment area of health service provider j (i.e., d_kj_≤d_0_);

A_i_ refers to the level of accessibility of mental health services for population centre i;

d_0_ is the service catchment size delineated by a travel time threshold;

d_ij_ is the estimated travel time between population centre i and health service provider j;

*G (*$d_{\begin{aligned} &ij \\ & \end{aligned}} , d_{0}$*)* is a Gaussian-based weight indicating the continuous distance decay between population centre i and health service provider j;

and $\beta$ is the distance decay coefficient denoting how health service accessibility varies with travel cost (i.e., travel distance or travel time).

Selecting appropriate service catchment sizes (d_0_) is critical, as they define the geographical scope of service availability for a population at a demand location. Overly restrictive or expansive catchment areas can substantially bias spatial accessibility measures [7, 8]. To assess the robustness of catchment sizes, we conducted a sensitivity analysis and tested four driving time thresholds: 15, 30, 45, and 60 minutes, informed by: (i) the 15-minute city concept [9-11], which prioritises proximity to essential services (e.g., healthcare) to enhance sustainability and resilience; (ii) extensions of the 15-minute city concept to 30-minute and 45-minute cities, which balance accessibility with social cohesion in lower-density contexts [12-14]; and (iii) the episodic nature of mental health service needs, which often entail a relatively longer travel time (e.g., 60 minutes) compared to routine care. This multi-threshold approach captures variability in accessibility across urban, peri-urban, and rural regions in SEQ while aligning with real-world travel behaviours. By testing thresholds from 15 to 60 minutes, we evaluated how accessibility patterns shift with varying definitions of travel time in the real world.

**References**

1. Luo W, Wang F. Measures of Spatial Accessibility to Healthcare in a GIS Environment: Synthesis and a Case Study in Chicago Region. Environment and Planning B: Urban Analytics and City Science. 2003;30(6):865-84.

2. Dai D. Black residential segregation, disparities in spatial access to health care facilities, and late-stage breast cancer diagnosis in metropolitan Detroit. Health & place. 2010;16(5):1038-52.

3. Wang L. Immigration, ethnicity, and accessibility to culturally diverse family physicians. Health & Place. 2007;13(3):656-71.

4. Wan N, Zhan FB, Zou B, Chow E. A relative spatial access assessment approach for analyzing potential spatial access to colorectal cancer services in Texas. Applied Geography. 2012;32(2):291-9.

5. Chen X, Jia P. A comparative analysis of accessibility measures by the two-step floating catchment area (2SFCA) method. International Journal of Geographical Information Science. 2019;33(9):1739-58.

6. Kwan MP. Space‐time and integral measures of individual accessibility: a comparative analysis using a point‐based framework. Geographical analysis. 1998;30(3):191-216.

7. Luo W, Whippo T. Variable catchment sizes for the two-step floating catchment area (2SFCA) method. Health & Place. 2012;18(4):789-95.

8. McGrail MR, Humphreys JS. Measuring spatial accessibility to primary health care services: Utilising dynamic catchment sizes. Applied Geography. 2014;54:182-8.

9. Moreno C, Allam Z, Chabaud D, Gall C, Pratlong F. Introducing the “15-Minute City”: Sustainability, Resilience and Place Identity in Future Post-Pandemic Cities. Smart Cities [Internet]. 2021; 4(1):[93-111 pp.].

10. Khavarian-Garmsir AR, Sharifi A, Sadeghi A. The 15-minute city: Urban planning and design efforts toward creating sustainable neighborhoods. Cities. 2023;132:104101.

11. Abbiasov T, Heine C, Sabouri S, Salazar-Miranda A, Santi P, Glaeser E, et al. The 15-minute city quantified using human mobility data. Nature Human Behaviour. 2024.

12. Levinson DM. The 30-minute city: Designing for access. Sydney, NSW, Australia: Network Design Lab; 2019.

13. Birkenfeld C, Victoriano-Habit R, Alousi-Jones M, Soliz A, El-Geneidy A. Who is living a local lifestyle? Towards a better understanding of the 15-minute-city and 30-minute-city concepts from a behavioural perspective in Montréal, Canada. Journal of Urban Mobility. 2023;3:100048.

14. Manifesty OR, Park JY. A Case Study of a 15-Minute City Concept in Singapore’s 2040 Land Transport Master Plan: 20-Minute Towns and a 45-Minute City. International Journal of Sustainable Transportation Technology. 2022;5(1):1-11.
