# Supplementary material for: Socio-spatial inequalities in accessibility of Indigenous community-controlled mental health services in South East Queensland, Australia
Source: Int J Health Geogr. 2025 Sep 26;24:24. doi: 10.1186/s12942-025-00415-9 (PMC12465140; doi:10.1186/s12942-025-00415-9)
Supplement: Supplementary file 2 — Additional file 2: Description of the bivariate global Moran’s I and bivariate local Moran’s I statistics. Details of the bivariate global Moran’s I and bivariate local Moran’s I statistics, including their application in this study. [file 12942_2025_415_MOESM2_ESM.docx]

**Additional file 2: Description of the bivariate global *Moran’s I* and bivariate local *Moran’s I* statistics**

Bivariate global *Moran’s I* provides a general overview of whether two variables exhibit spatial autocorrelation across the entire dataset. It has values ranging from -1 to 1. A positive value indicates that high levels of accessibility are spatially correlated with high values of a socio-demographic factor for SEQ; A negative value indicates that high levels of accessibility are spatially correlated with low values of a socio-demographic factor for SEQ; A value close to zero suggests no spatial autocorrelation between the two variables for SEQ. We also conducted a permutation test to assess the statistical significance of the bivariate global *Moran’s I* values, with pseudo-significance set at the *p*<0.1, *p*<0.05, and *p*<0.01 levels [1].

Bivariate local *Moran’s I* examines the local associations between accessibility and each of the three socio-demographic factors for each individual SA2, identifying specific SA2s where the spatial correlation between two variables is particularly either strong or weak [2]. The values of bivariate local *Moran’s I* range between -1 and 1, with a positive value implying that SA2s with high accessibility values are surrounded by SA2s with high values of a socio-demographic factor, or SA2s with low accessibility values are surrounded by SA2s with low values of a socio-demographic factor. Conversely, a negative value of bivariate local *Moran’s I* reflects SA2s with high accessibility values are surrounded by SA2s with low values of a socio-demographic factor, or SA2s with low accessibility values are surrounded by SA2s with high values of a socio-demographic factor [3]. The strength of spatial associations between accessibility and socio-demographic factors increases with a higher absolute value of the bivariate local *Moran’s I*. The criterion for statistical significance of the bivariate local *Moran’s I* value in the bivariate LISA map is set at the *p*<0.5 level.

In a bivariate LISA map, areas with socio-spatial inequalities are identified by interpreting the spatial patterns of accessibility associated with each socio-demographic factor, which can be categorised into five types: High-High indicates high accessibility values surrounded by high values of a particular socio-demographic factor; High-Low indicates high accessibility values surrounded by low values of the socio-demographic factor; Low-High indicates low accessibility values surrounded by high values of the socio-demographic factor; Low-Low indicates low accessibility values surrounded by low values of the socio-demographic factor; and non-significant indicates that no spatial association was identified as statistically significant for these locations. It is worth noting that the interpretation of these association types should consider the way the socio-demographic variables are measured. For instance, a Low-Low cluster between accessibility and Indigenous population proportion would mean that areas with low levels of accessibility are clustered with areas with a low concentration of Indigenous Australians in SEQ, but a Low-Low cluster between accessibility and IRSAD score would mean areas with low levels of accessibility are clustered with areas with greater levels of social disadvantage, given that a lower IRSAD score indicates a higher level of social disadvantage.

**References**

1. Cheng Z. The spatial correlation and interaction between manufacturing agglomeration and environmental pollution. Ecological Indicators. 2016;61:1024-32.

2. Jin T, Cheng L, Wang K, Cao J, Huang H, Witlox F. Examining equity in accessibility to multi-tier healthcare services across different income households using estimated travel time. Transport Policy. 2022;121:1-13.

3. Sun Y, Hu X, Xie J. Spatial inequalities of COVID-19 mortality rate in relation to socioeconomic and environmental factors across England. Science of The Total Environment. 2021;758:143595.
