# Supplementary material for: Socio-spatial inequalities in accessibility of Indigenous community-controlled mental health services in South East Queensland, Australia
Source: Int J Health Geogr. 2025 Sep 26;24:24. doi: 10.1186/s12942-025-00415-9 (PMC12465140; doi:10.1186/s12942-025-00415-9)
Supplement: Supplementary file 3 — Additional file 3: Spatial distribution of the socio-economic disadvantage in SEQ. Maps of the 2021 IRSAD and IRSEO score distributions by SA2 across SEQ [file 12942_2025_415_MOESM3_ESM.docx]

**Additional File 4:** **Focus areas for prioritising Indigenous community-controlled mental health service development in South-East Queensland (SEQ)**

To synthesise the findings from the accessibility maps and socio-spatial inequality analysis, we developed a summary table of identified focus areas by SA2, alongside their socio-demographic characteristics. These including the Index of Relative Socio-economic Advantage and Disadvantage (IRSAD) and the Indigenous Relative Socio-Economic Outcomes (IRSEO) (Table 1). The focus areas, highlighted in Fig. 4 of the manuscript, are categorised into three types: (1) *Low Access* ***–****High Indigenous Proportion* refers to areas where SA2s with relatively low levels of accessibility and relatively high Indigenous population proportions are clustered; (2) *Low Access* ***–****High IRSEO Disadvantage* denotes areas where SA2s with relatively low levels of accessibility and relatively high levels of Indigenous-specific socio-economic disadvantage are clustered; and (3) *Low Access* ***–****High IRSAD Disadvantage* indicates areas where SA2s with relatively low levels of accessibility and relatively high levels of socio-economic disadvantage for the total population are clustered.

Table 1. List of identified focus areas by SA2 and their socio-demographic characteristics

| **SA2 Name** | **Accessibility within a 30-min driving time (Mean=1.04)** | **Total population (Number, Mean=10,618)** | **Proportion of Indigenous populations (%, Mean=2.8)** | **IRSAD score (Mean = 1,010)** | **IRSEO score (Mean = 2.2)** | **Focus area type** |
| --- | --- | --- | --- | --- | --- | --- |
| Crows Nest - Rosalie | 0.00 | 9175 | 3.47 | 924 | 3.16 | Low Access – High Indigenous Proportion |
| Broadbeach Waters | 0.84 | 8155 | 0.95 | 1076 | 2.86 | Low Access – High IRSEO Disadvantage |
| Mermaid Beach - Broadbeach | 1.02 | 13996 | 1.73 | 1039 | 2.86 | Low Access – High IRSEO Disadvantage |
| Arundel | 0.62 | 11166 | 2.02 | 1002 | 2.86 | Low Access – High IRSEO Disadvantage |
| Biggera Waters | 0.38 | 9970 | 1.80 | 972 | 2.86 | Low Access – High IRSEO Disadvantage |
| Coombabah | 0.38 | 10300 | 1.85 | 936 | 2.86 | Low Access – High IRSEO Disadvantage |
| Labrador | 0.50 | 18647 | 4.01 | 930 | 2.86 | Low Access – High IRSEO Disadvantage |
| Paradise Point - Hollywell | 0.46 | 9994 | 0.90 | 1045 | 2.86 | Low Access – High IRSEO Disadvantage |
| Runaway Bay | 0.42 | 9304 | 1.14 | 1012 | 2.86 | Low Access – High IRSEO Disadvantage |
| Tamborine - Canungra | 0.00 | 15696 | 2.87 | 1021 | 2.86 | Low Access – High IRSEO Disadvantage |
| Currumbin Valley - Tallebudgera | 0.80 | 7663 | 1.51 | 1055 | 2.86 | Low Access – High IRSEO Disadvantage |
| Mudgeeraba - Bonogin | 0.91 | 19548 | 3.71 | 1036 | 2.86 | Low Access – High IRSEO Disadvantage |
| Carrara | 0.82 | 13151 | 2.52 | 1002 | 2.86 | Low Access – High IRSEO Disadvantage |
| Highland Park | 0.88 | 8431 | 2.36 | 983 | 2.86 | Low Access – High IRSEO Disadvantage |
| Nerang - Mount Nathan | 0.69 | 21251 | 6.07 | 965 | 2.86 | Low Access – High IRSEO Disadvantage |
| Pacific Pines - Gaven | 0.58 | 18303 | 3.50 | 1006 | 2.86 | Low Access – High IRSEO Disadvantage |
| Worongary - Tallai | 0.79 | 8539 | 1.54 | 1044 | 2.86 | Low Access – High IRSEO Disadvantage |
| Coomera | 0.67 | 20227 | 6.26 | 993 | 2.86 | Low Access – High IRSEO Disadvantage |
| Helensvale | 0.59 | 18942 | 3.28 | 1029 | 2.86 | Low Access – High IRSEO Disadvantage |
| Hope Island | 0.54 | 14667 | 1.55 | 1063 | 2.86 | Low Access – High IRSEO Disadvantage |
| Jacobs Well - Alberton | 0.25 | 4603 | 1.05 | 993 | 2.86 | Low Access – High IRSEO Disadvantage |
| Oxenford - Maudsland | 0.64 | 17922 | 3.92 | 1011 | 2.86 | Low Access – High IRSEO Disadvantage |
| Ormeau (East) - Stapylton | 0.65 | 10677 | 3.30 | 989 | 2.86 | Low Access – High IRSAD Disadvantage |
| Pimpama - North | 0.70 | 9923 | 3.04 | 996 | 2.86 | Low Access – High Indigenous Proportion, Low Access – High IRSEO Disadvantage |
| Upper Coomera (South) - Wongawallan | 0.50 | 11417 | 2.53 | 1043 | 2.86 | Low Access – High IRSEO Disadvantage |
| Upper Coomera - North | 0.73 | 20291 | 6.14 | 975 | 2.86 | Low Access – High IRSEO Disadvantage |
| Willow Vale - Pimpama (West) | 0.72 | 7021 | 2.04 | 1003 | 2.86 | Low Access – High IRSEO Disadvantage |
| Clear Island Waters | 0.72 | 4402 | 0.38 | 1067 | 2.86 | Low Access – High IRSEO Disadvantage |
| Merrimac | 1.02 | 7205 | 1.11 | 986 | 2.86 | Low Access – High IRSEO Disadvantage |
| Ashmore | 0.60 | 12422 | 1.85 | 1007 | 2.86 | Low Access – High IRSEO Disadvantage |
| Molendinar | 0.61 | 6443 | 1.54 | 991 | 2.86 | Low Access – High IRSEO Disadvantage |
| Parkwood | 0.54 | 8839 | 2.25 | 1017 | 2.86 | Low Access – High IRSEO Disadvantage |
| Southport - North | 0.60 | 17894 | 3.64 | 954 | 2.86 | Low Access – High IRSEO Disadvantage |
| Southport - South | 0.59 | 18890 | 3.65 | 981 | 2.86 | Low Access – High IRSEO Disadvantage |
| Benowa | 0.63 | 9886 | 0.89 | 1053 | 2.86 | Low Access – High IRSEO Disadvantage |
| Bundall | 0.73 | 4897 | 0.76 | 1068 | 2.86 | Low Access – High IRSEO Disadvantage |
| Main Beach | 0.53 | 4006 | 0.38 | 1064 | 2.86 | Low Access – High IRSEO Disadvantage |
| Surfers Paradise - North | 0.64 | 12199 | 1.44 | 999 | 2.86 | Low Access – High IRSEO Disadvantage |
| Surfers Paradise - South | 0.65 | 14213 | 1.40 | 993 | 2.86 | Low Access – High IRSEO Disadvantage |
| Forest Lake - Ellen Grove | 0.87 | 22480 | 3.91 | 995 | 2.68 | Low Access – High IRSAD Disadvantage |
| Boonah | 0.00 | 12616 | 2.92 | 958 | 1.59 | Low Access – High Indigenous Proportion |
| Lowood | 0.07 | 14203 | 7.45 | 912 | 0.53 | Low Access – High Indigenous Proportion, Low Access – High IRSAD Disadvantage |
| Rosewood | 0.48 | 14218 | 8.42 | 934 | 0.87 | Low Access – High Indigenous Proportion, Low Access – High IRSAD Disadvantage |
| Brassall | 0.49 | 12098 | 8.04 | 908 | 0.87 | Low Access – High IRSAD Disadvantage |
| Bundamba | 0.67 | 9339 | 5.70 | 895 | 0.87 | Low Access – High Indigenous Proportion, Low Access – High IRSAD Disadvantage |
| Churchill - Yamanto | 0.43 | 6809 | 4.06 | 926 | 0.87 | Low Access – High Indigenous Proportion, Low Access – High IRSAD Disadvantage |
| Ipswich - Central | 0.48 | 6718 | 4.22 | 923 | 0.87 | Low Access – High Indigenous Proportion, Low Access – High IRSAD Disadvantage |
| Ipswich - East | 0.59 | 17981 | 9.98 | 895 | 0.87 | Low Access – High IRSAD Disadvantage |
| Leichhardt - One Mile | 0.38 | 8268 | 8.20 | 830 | 0.87 | Low Access – High IRSAD Disadvantage |
| North Ipswich - Tivoli | 0.56 | 6418 | 3.56 | 904 | 0.87 | Low Access – High Indigenous Proportion, Low Access – High IRSAD Disadvantage |
| Raceview | 0.51 | 15158 | 7.95 | 932 | 0.87 | Low Access – High Indigenous Proportion, Low Access – High IRSAD Disadvantage |
| Camira - Gailes | 0.75 | 9249 | 4.41 | 939 | 0.87 | Low Access – High IRSAD Disadvantage |
| Collingwood Park - Redbank | 0.65 | 11682 | 4.63 | 924 | 0.87 | Low Access – High IRSAD Disadvantage |
| Redbank Plains | 0.52 | 24347 | 13.68 | 884 | 0.87 | Low Access – High IRSAD Disadvantage |
| Beenleigh | 0.86 | 8426 | 4.00 | 856 | 1.00 | Low Access – High IRSAD Disadvantage |
| Edens Landing - Holmview | 0.80 | 9544 | 3.68 | 945 | 1.00 | Low Access – High IRSAD Disadvantage |
| Mount Warren Park | 0.77 | 5741 | 1.79 | 934 | 1.00 | Low Access – High IRSAD Disadvantage |
| Boronia Heights - Park Ridge | 0.91 | 19165 | 6.97 | 924 | 1.00 | Low Access – High IRSAD Disadvantage |
| Bethania - Waterford | 1.02 | 12132 | 4.89 | 911 | 1.00 | Low Access – High IRSAD Disadvantage |
| Beachmere - Sandstone Point | 0.38 | 16475 | 6.34 | 923 | 0.55 | Low Access – High Indigenous Proportion |
| Bribie Island | 0.14 | 20619 | 5.43 | 927 | 0.55 | Low Access – High Indigenous Proportion, Low Access – High IRSAD Disadvantage |
| Elimbah | 0.88 | 4286 | 1.24 | 989 | 0.55 | Low Access – High Indigenous Proportion, Low Access – High IRSAD Disadvantage |
| Wamuran | 0.74 | 4225 | 1.21 | 992 | 0.55 | Low Access – High Indigenous Proportion, Low Access – High IRSAD Disadvantage |
| Noosa Heads | 0.00 | 5120 | 0.29 | 1063 | 2.88 | Low Access – High IRSEO Disadvantage |
| Noosaville | 0.00 | 9331 | 0.87 | 1026 | 2.88 | Low Access – High IRSEO Disadvantage |
| Sunshine Beach | 0.00 | 6854 | 0.63 | 1055 | 2.88 | Low Access – High IRSEO Disadvantage |
| Tewantin | 0.00 | 10544 | 2.13 | 959 | 2.88 | Low Access – High IRSEO Disadvantage |
| Peregian Beach - Marcus Beach | 0.00 | 4112 | 0.50 | 1066 | 2.88 | Low Access – High IRSEO Disadvantage |
| Noosa Hinterland | 0.00 | 24216 | 4.56 | 998 | 2.88 | Low Access – High IRSEO Disadvantage |
| Gowrie (Qld) | 0.44 | 7060 | 3.28 | 990 | 3.16 | Low Access – High Indigenous Proportion |
| Highfields | 0.54 | 14574 | 4.05 | 1051 | 3.16 | Low Access – High Indigenous Proportion |
