# Supplementary material for: Socio-spatial inequalities in accessibility of Indigenous community-controlled mental health services in South East Queensland, Australia
Source: Int J Health Geogr. 2025 Sep 26;24:24. doi: 10.1186/s12942-025-00415-9 (PMC12465140; doi:10.1186/s12942-025-00415-9)
Supplement: Supplementary file 4 — Additional file 4: Focus areas for prioritising Indigenous community-controlled mental health service development in SEQ. The summary table of identified focus areas by SA2, alongside their socio-demographic characteristics [file 12942_2025_415_MOESM4_ESM.docx]

**Additional File 3: Spatial distribution of the socio-economic disadvantage in South-East Queensland (SEQ)**


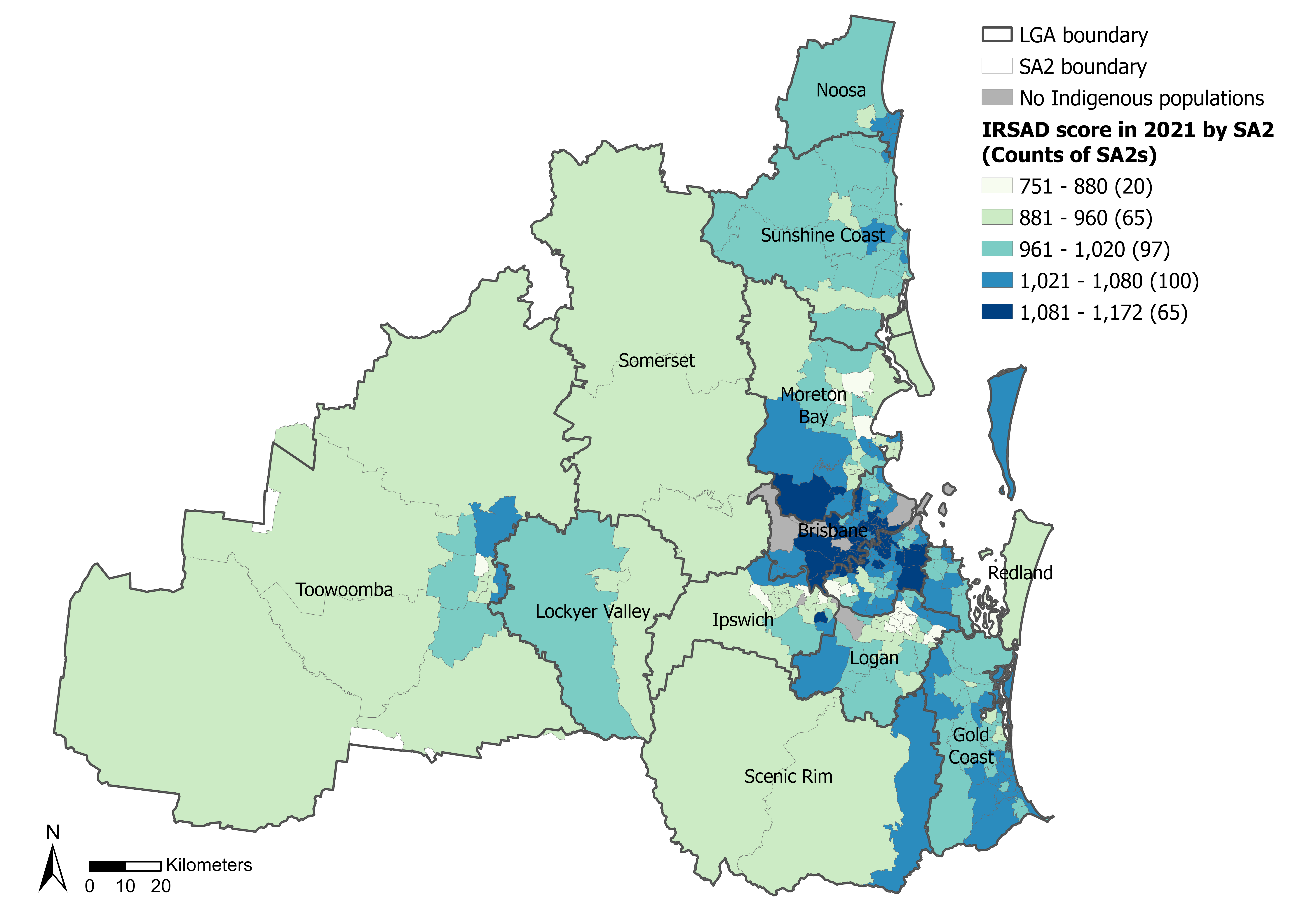


Fig. A. Distribution of the Index of Relative Socio-economic Advantage and Disadvantage (IRSAD) in 2021 across SEQ (Classification method: Natural Breaks (Jenks))


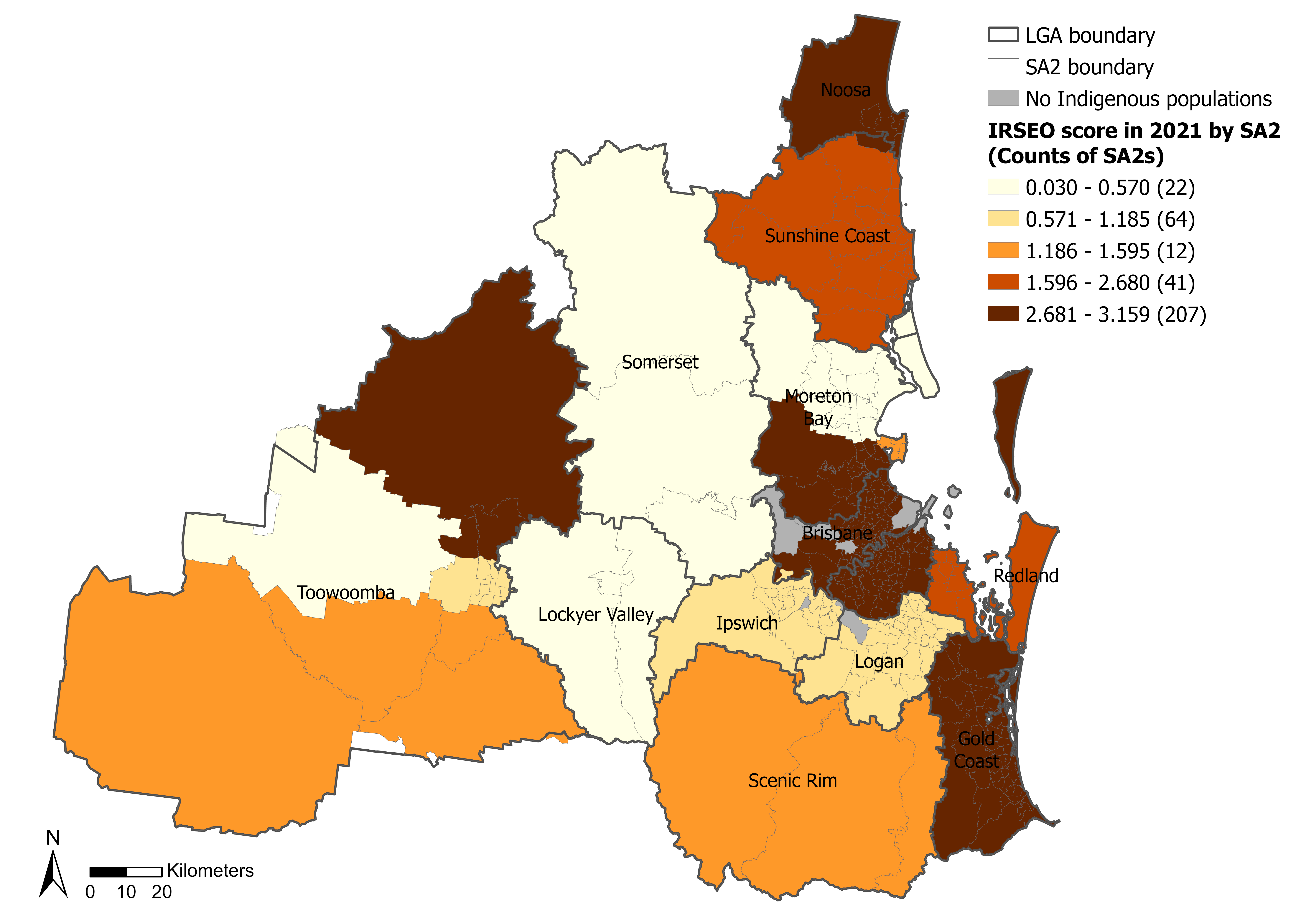


Fig. B. Distribution of the Indigenous Relative Socio-Economic Outcomes (IRSEO) scores in 2021 across SEQ (Classification method: Natural Breaks (Jenks))
